# Increased cortical neuronal responses to NMDA and improved attentional set-shifting performance in rats following prebiotic (B-GOS^®^) ingestion

**DOI:** 10.1016/j.euroneuro.2017.11.001

**Published:** 2018-01

**Authors:** Benjamin Gronier, Helene M. Savignac, Mathieu Di Miceli, Sherif M. Idriss, George Tzortzis, Daniel Anthony, Philip W.J. Burnet

**Affiliations:** a Leicester School of Pharmacy, De Montfort University, The Gateway, Leicester LE1 9BH, UK; b 4D Pharma, Cornhill Road, Aberdeen AB25 2ZS, UK; c Center of Brain, Behavior and Metabolism (CBBM), University of Lubeck, 23562 Lubeck, Germany; d Clasado Biosciences Ltd., Reading RG6 6BZ, UK; e Department of Pharmacology, University of Oxford, Oxford OX1 3QT, UK; f Department of Psychiatry, University of Oxford, Warneford Hospital, Oxford OX3 7JX, UK

**Keywords:** Electrophysiology, Cognition, Short-chain fatty acid, Microbiota, Glutamate

## Abstract

We have previously shown that prebiotics (dietary fibres that augment the growth of indigenous beneficial gut bacteria) such as Bimuno^™^ galacto-oligosaccharides (B-GOS^®^), increased N-methyl-D-aspartate (NMDA) receptor levels in the rat brain. The current investigation examined the functional correlates of these changes in B-GOS^®^-fed rats by measuring cortical neuronal responses to NMDA using in vivo NMDA micro-iontophoresis electrophysiology, and performance in the attentional set-shifting task. Adult male rats were supplemented with B-GOS^®^ in the drinking water 3 weeks prior to in vivo iontophoresis or behavioural testing. Cortical neuronal responses to NMDA iontophoresis, were greater (+30%) in B-GOS^®^ administered rats compared to non-supplemented controls. The intake of B-GOS^®^ also partially hindered the reduction of NMDA responses by the glycine site antagonist, HA-966. In the attentional set-shifting task, B-GOS^®^ -fed rats shifted from an intra-dimensional to an extra-dimensional set in fewer trials than controls, thereby indicating greater cognitive flexibility. An initial exploration into the mechanisms revealed that rats ingesting B-GOS^®^ had increased levels of plasma acetate, and cortical GluN2B subunits and Acetyl Co-A Carboxylase mRNA. These changes were also observed in rats fed daily for 3 weeks with glyceryl triacetate, though unlike B-GOS^®^, cortical histone deacetylase (HDAC1, HDAC2) mRNAs were also increased which suggested an additional epigenetic action of direct acetate supplementation. Our data demonstrate that a pro-cognitive effect of B-GOS^®^ intake in rats is associated with an increase in cortical NMDA receptor function, but the role of circulating acetate derived from gut bacterial fermentation of this prebiotic requires further investigation.

## 1 Introduction

The link between enteric microbiota and brain function is now widely accepted, has been considered as ‘a paradigm shift in neuroscience’ (Mayer et al., 2014). Mice depleted of microbiota (germ-free mice), display altered behaviours and reduced levels of Brain Derived Neurotrophic Factor (BDNF) and N-methyl-D-aspartate receptors (NMDARs), which are crucial for cognitive function (Sudo et al., 2004). Conversely, gut microbiota enrichment with specific probiotics, live beneficial bacteria, or prebiotics, indigestible compounds that augment the growth of intrinsic beneficial microbes, improve cognitive performance in rodents (Savignac et al., 2015, Vázquez et al., 2015, O'Hagan et al., 2017) and humans (Schmidt et al., 2015, Steenbergen et al., 2015). The reduction of attentional bias to negative emotional stimuli in healthy volunteers following a dietary supplementation with the Bimuno^™^ galacto-oligosaccharide (B-GOS^®^) prebiotic (Schmidt et al., 2015), may be a corollary of increased levels of NMDAR subunits which we have observed in the rat frontal cortex following B-GOS feeding (Savignac et al., 2013). However, the functional correlates of these changes have not been explored.

The prebiotic properties of B-GOS^®^ have been extensively studied, and the product which contains a mixture of galacto-oligosaccharides of several lengths (2–7 saccharides) has been consistently shown to selectively increase *Bifidobacteria* and, to some extent, *Lactobacilli* in both humans and animals (Tzortzis et al., 2005, Depeint et al., 2008, Vulevic et al., 2008, Silk et al., 2009, Savignac et al., 2013). Understanding how B-GOS^®^ modulates NMDARs has important implications for both the prevention of the age-related decline in cognitive function (Nicolle and Baxter 2003), and the treatment of neuropsychiatric disorders such as schizophrenia, where aberrant glutamate neurotransmission and cognitive deficits cannot be rescued by conventional medication. Given that the loss of NMDAR function impairs cellular responses in the rodent cortex (Rompala et al., 2013), we hypothesise that the elevated cortical GluN1 and D-serine following B-GOS^®^ ingestion (Savignac et al., 2013), increases cortical NMDAR-mediated neural activity, and related behaviours. In the latter instance, based on existing data, increased cortical NMDAR function in healthy animals may improve attentional set-shifting performance (cognitive flexibility), a prefrontal cortex (PFC)-dependent behaviour often impaired by NMDAR antagonists (Neill et al., 2010, Wallace et al., 2014), or the natural decrease in cortical NMDAR levels during aging (Nicolle and Baxter, 2003, Rodefer and Nguyen, 2008). However, there are no studies demonstrating improved cognitive flexibility in experimentally naïve rodents following an elevation of NMDARs, and/or their function, in the PFC. The increase of central NMDAR subunits in the rat brain following B-GOS ingestion, provides a model to test this.

There is also an urgent need to ascertain the mechanisms that underlie the central effects of B-GOS^®^, so that key intermediaries of microbe-brain interactions can be revealed. The short-chain fatty acids (SCFAs) that arise from bacterial fermentation of dietary carbohydrates in the host gut, have been suggested to be one such mediator (Sarkar et al., 2016). Fermentation of B-GOS has been shown to produce significant amounts of acetate, and moderate amounts of butyrate (Grimaldi et al., 2016, Grimaldi et al., 2017). Both of these SCFAs can have central effects, particularly at the epigenetic level where they both influence the expression of brain histone deacetylases (HDACs) (Soliman et al., 2012, Han et al., 2014), but a link between metabolic acetate and NMDARs has also been demonstrated (Hirose et al., 2009, Singh et al., 2016). In this regard, if acetate is involved in B-GOS^®^ mediated changes of NMDAR function, then direct acetate supplementation might be expected to have similar effects on central NMDAR levels as the prebiotic itself.

The aim of the current study was to, first, confirm with in vivo iontophoresis electrophysiology increased NMDAR function in the rat frontal cortex following B-GOS^®^ intake alone, or in the presence of the NMDAR glycine-site antagonist, HA-966. Second, test if administration of B-GOS^®^ facilitated cognitive flexibility, based on the prebiotic-mediated elevation of cortical NMDARs. Third, explore the role of acetate in the actions of B-GOS^®^ by (i) measuring plasma and brain levels of this SCFA in B-GOS^®^ -fed and control rats, and (ii) quantifying in all animals the expression of frontal cortex Acetyl Co-enzyme A Carboxylase (ACC) mRNA. Earlier work has demonstrated that a single systemic injection of acetate into rats increases the activity of hypothalamic ACC (Frost et al., 2014). This enzyme metabolises the Acetyl Co-enzyme A that is produced from the acetate sequestered in tissues. The abundance of cortical ACC mRNA therefore, was used as an indicator of acetate metabolism. In addition, the expression of HDAC(1–4) genes in the cortex were measured to evaluate whether B-GOS^®^, via acetate, affects epigenetic processes. Finally, plasma and brain acetate, cortical GluN subunit levels, and ACC and HDAC(1–4) mRNAs were evaluated in glyceryl triacetate fed rats to further examine the potential involvement of acetate in NMDAR modulation.

## 2 Experimental procedures

### 2.1 Animals

Adult male Sprague Dawley rats (250–300 g) were housed (3rats/cage) in standard laboratory conditions (21±1 °C; lights on 07:00–19:00 h), with free access to water and standard laboratory chow. For behavioural testing, rats had limited access to standard chow (food restricted) and maintained at 85–90% of their free-feeding weight throughout experiments. The nutritional content of the standard diet provided (Envigo, USA, product no. 2916) included protein (16%), fat (4%), crude fibre (3.3%), and insoluble fibre (eg cellulose, 15%). All procedures were carried out in accordance with UK Home Office Animals (Scientific Procedures) Act (1986) and associated Home Office guidelines. The procedures specific to this study were approved by the local Animal Welfare and Ethical Review Body (AWERB) at both DeMontfort and Oxford Universities.

### 2.2 Prebiotic administration

Rats received normal drinking water, drinking water supplemented with 3% B-GOS^®^, or drinking water supplemented with B-GOS^®^ free sugars (BFS: lactose [7.5 g/L], glucose [4.2 g/L] and galactose [3.6 g/L]) for 3 weeks and throughout behavioural tests. The inclusion of a BFS group was to determine whether any behavioural effects of B-GOS^®^ was attributable to the ingestion of the sugars in the prebiotic mixture, rather than the galato-oligosaccharides. Body weight and fluid intake in all groups were recorded every 2 days. Food restriction for behavioural studies commenced 7 days after the provision of prebiotic.

### 2.3 Glyceryl triacetate administration

In a separate study male rats (250–300 g) were gavaged with glyceryl triacetate (GTA, 1 g/ml, Sigma Aldrich, UK) at a final dose of 3 g/kg, or the same volume of water alone (control) daily for 3 weeks. The choice of GTA dose and control was based on a previous studies (Singh et al., 2016).

### 2.4 In vivo iontophoresis electrophysiology

These experiments were conducted on B-GOS^®^-fed rats and water controls as previously described (Di Miceli and Gronier, 2015). Animals were deeply anaesthetised with urethane (1.2–1.7 g/kg, intraperitoneal, with additional doses if necessary), secured to a stereotaxic frame and maintained at 36–37 °C. Briefly, under urethane anaesthesia, animals underwent stereotaxic surgery to drill a hole in the skull over the PFC (co-ordinates from Bregma: anteroposterior + 2.5–3.7 mm, lateral 0.3–2 mm). Five-barrel glass micropipettes (ASI, USA), pulled on a PP-830 electrode puller (Narishige, Japan) were used. The central barrel, filled with a saline solution, was used for recording, and the four-side barrels were filled with at least one of the following solutions: NMDA (30 mM in 200 mM of NaCl, pH 7.5), HA-966 (10 mM, in 200 mM NaCl, pH 4) and 1 M NaCl for current balancing (later found to be unnecessary).

Outputs from the electrode were sent to a Neurolog AC pre-amplifier and amplifier (Digitimer, UK). Signals were filtered and sent to an audio amplifier, a digital storage oscilloscope and a 1401 interface connected to a computer running Spike 2 (CED, Cambridge, UK) for data capture and analysis. Descent of the electrode was accomplished using a hydraulic micromanipulator (Narishige). Coordinates for the PFC were as follows: anteroposterior 2.5–3.7 mm, lateral 0.3–2 mm, 1.5–5 mm below cortical surface. Neurons were identified according to previous electrophysiological criteria established from studies carried on formally identified pyramidal neurons (Hajos et al., 2003, Puig et al., 2005, Tseng et al., 2006, Kargieman et al., 2007, Gronier, 2011, Wang et al., 2011): a broad action potential (>1 ms), with a biphasic or triphasic, large waveform, starting with a positive inflection, a slow firing rate typically between 1 and 50 spikes/10 s and irregular firing pattern, often with burst activity.

Neuronal response was measured by the software (Spike 2, Cambridge Electronic design) as actions potentials (number of spikes) generated both at baseline and in response to drugs injections, from a neuron per 10 seconds. All data were expressed as a net response relative to baseline recordings. NMDA or AMPA were ejected as cations by applying a negative current at a given intensity (−1 to 15 nA) for 40–70 s at 70–100 s interval. HA-966 was ejected as an anion by applying a positive current (−3 to 35 nA) during a long-lasting stable (>200 s) neuronal activation elicited by NMDA or AMPA application. Drugs were retained in the iontophoretic channel by applying a low intensity current (2–5 Na) of opposite charge of the ejection current.

### 2.5 Attentional set-shifting task (ASST)

This task was carried out on B-GOS^®^ supplemented rats and water controls using an established procedure (Burnham et al., 2010). The testing chamber was a home cage with Perspex dividers separating the cage into a start chamber and two identically sized choice chambers with access controlled by Perspex doors. Ceramic bowls were placed within the choice chambers and baited with pieces of sweetened cereal (Honey Nut Cheerios, Nestle, Surrey, UK). A piece of cereal was also placed at the bottom of each bowl to mask the odour of the bait, and was protected by a fixed wire mesh to make it inaccessible to rats. During testing, one bowl was baited and rats discriminated the baited bowl using the texture and/or the odour of the digging medium. Choice was defined as active digging or foraging with the snout in the digging medium. Rats were habituated to the apparatus and initially taught to dig in baited bowls filled with home cage bedding. On the day prior to testing, rats performed two simple discriminations, one based on odour (lavender versus lemon scented bedding) and one based on medium (small wooden beads versus large wooden beads), to a criterion of six consecutive correct trials. The order of training discriminations (odour/medium) and the rewarded odour and digging medium were counter-balanced, and exemplars (Table 1) were not used again throughout testing.

**Table 1:** Discrimination phases of the attentional set-shifting task and exemplars. Rewarded exemplars are underlined.

Discrimination	Odour pair	Medium pair
Simple discrimination	cinnamon/cumin	Bedding
Compound discrimination	cinnamon/cumin	cat litter/ground cat litter
Reversal 1	cinnamon/cumin	cat litter/ground cat litter
Intra-dimensional shift	clove/thyme	pebbles/gravel
Reversal 2	clove/thyme	pebbles/gravel
Extra-dimensional shift	mint/paprika	sawdust/shavings
Reversal 3	mint/paprika	sawdust/shavings

All the discriminations in the testing sequence were presented in a single test day. The first four trials of each discrimination were “discovery” trials, during which the rat was allowed to dig in medium both bowls, to discover which bowl contained the reward. An error was recorded if the rat dug first in the unbaited bowl, but these trials were not included in the trials or errors to criterion score. After the first four trials, if the rat dug in the unbaited bowl, an error was recorded and access to the correct bowl was denied. The rat was allowed to return to the start chamber of its own accord to start the next trial. Testing continued until the rat reached a criterion of six correct consecutive trials. The sequence and composition of several discriminations used in the test are summarized in Table 1. A simple discrimination (SD), between either two odours or two digging mediums, was followed by a compound discrimination (CD) which had the same positive stimulus as the SD, but included a new irrelevant dimension which did not predict the location of the reward. This was followed a reversal of the CD (R1) where all of the stimuli remained the same, and the relevant dimension remained the same, but the rewarded and non-rewarded stimuli within the dimension were reversed. The rats were then subjected to an Intra-dimensional (ID) shift which was a CD in which both the relevant and irrelevant stimuli changed, but the rewarded dimension (either odour or medium) remained the same. This was followed by a second reversal (R2) stage as described for R1. The rats were then exposed to an extra-dimensional (ED) shift, where the relevant and irrelevant stimuli changed, as well as the relevant dimension, so if odour had previously predicted the location of the reward, the digging would become the relevant dimension. A third reversal (R3) followed the ED, as described for R1 and R2. The experimenter was blind to treatment and remained in the experimental room silently during experiments for live scoring. Animals were tested in a controlled, random fashion regarding cage and treatment groups, and then were returned to their home cage where provision of prebiotic or control solutions continued. All rats were humanely culled 24 h after behavioural testing.

### 2.6 Blood and tissue collection

On completion of all experiments rats were culled and the trunk blood collected in sterile 2 ml heparin-coated Eppendorf tubes. Samples were centrifuged for 5 min in a bench-top centrifuge at maximum speed, the plasma was removed and stored at −80 °C prior to acetate assays. The frontal cortex was cut from cerebrum, bisected and stored at −80 °C prior to use. Faecal pellets were also collected from all rats that had been subjected to the ASST, and frozen at −80 °C prior to DNA extraction.

### 2.7 Western blotting

One hemisphere of the frontal cortex from each group of rats was homogenised in RIPA buffer (Sigma-Aldrich, UK), and run on western blots as previously described (Savignac et al., 2013). Equal concentrations of protein extracts of the cortex (10 ug) from all groups (*n*=8 rats/group) were mixed with loading buffer (50 mM 1, 4-dithiothreitol and 0.025% bromophenol blue), and fractionated with a molecular weight marker (GE Healthcare, Buckinghamshire, UK) by electrophoresis on pre-cast 7.5% SDS/polyacrylamide gels (Biorad,UK), and trans-blotted onto polyvinyl difluoride (PVDF) membranes (Immobilon-P, Millipore, Watford, UK).

The membranes were blocked with 5% (w/v) non-fat milk in PBS containing 0.1% Tween^20^ (PBST) for 45 min, and then incubated for 1 h at room temperature in incubation buffer (PBST with 2% [w/v] milk) containing a primary antibody (diluted 1:1000) against one of three NMDAR subunits: GluN1 (AB9864, Millipore, UK), GluN2A (AB1555, Millipore, UK) and GluN2B (AB15362, Millipore, UK), and β-actin (Sigma-Aldrich, UK, diluted 1:500,000). Membranes were then washed three times for ten minutes in PBST and incubated for 30 min in HRP-linked secondary antibody in blocking buffer. Immunoreactive bands were visualized by chemiluminescence using the ECL-Plus kit (GE Healthcare, Buckinghamshire, UK), and apposing membranes to X-ray film (Kodak BioMax AR film). All antibodies produced a single band of expected molecular weight. The optical densities (OD) of bands were measured using the AlphaImager 3400, and the data expressed as OD ratios of NMDAR subunit:β-actin.

### 2.8 Extraction of cortical RNA and faecal DNA, and quantitative PCR (QPCR)

Total RNA was extracted from the remaining fragment of cortical tissue using Tri-Reagent according to manufacturer's instructions, and reverse-transcribed to cDNA, using a commercial kit (Life Technologies, UK). The SYBR Green methodology (Power SYBER, Life Technologies, UK) was used to quantify the expression of mRNAs encoding Acetyl Co-Enzyme-A Carboxylase (ACC) and HDAC(1-4) in all cortical cDNA preparations. The amplification of ACC was achieved using forward (5'-GCT GAA GTC CCT GGA TCA CC-3') and reverse (5'-CTT TTA CAG CAC ACT GTT CC-3') primers that spanned 1012-1228 base-pairs of the rat ACC gene (NM_022193.1). QPCR of HDAC(1-4) mRNAs used previously published primers (Han et al, 2014).

Bacteria DNA from faecal samples was extracted using QIAamp DNA Stool Mini Kit (Qiagen, Hilden, Germany) after mechanical disruption. The abundance of 16S DNA encoding specific microbial genera and total bacteria in faecal pellets were analysed using qPCR and previously published primers (Morel et al., 2015). The SYBR Green methodology was used to amplify 20 ng DNA. All QPCR assays were performed on a 7900HT Fast Real-Time PCR System (Applied Biosystems, USA).

### 2.9 Acetate assay

The concentration of acetate in plasma and cortical homogenates were measured using a commercial colorimetric assay (ab204719, Abcam, UK). Prior to all assays, plasma and RIPA extracts of frontal cortex (see above) were thawed and 50 μl were aliquoted into1.5 ml Eppendorf tubes. An equal volume of isopropanol was then added to all samples and thoroughly mixed to precipitate proteins. Tubes were centrifuged on a bench-top centrifuge for 5 min at maximum speed, and the supernatant was transferred to a fresh tube. All samples were dried down and the anhydrous metabolites dissolved in 50 μl acetate buffer. Ten microliters of protein-free plasma and cortical extracts were assayed for acetate in duplicate with a standard curve according to manufacturer's instructions.

### 2.10 Data analysis

Normal distribution of data was confirmed using Shapiro-Wilk test (SPSS, ver.22). All data are expressed as mean ± standard error of the mean (SEM). Electrophysiological, behavioural body weight and fluid intake data were analysed with Two-way ANOVA (repeated measures), and Bonferroni *post-hoc* correction. All QPCR data are presented as fold change (relative to GAPDH for tissue, and total bacterial 16S for faecal DNA) which were calculated using the 2^−ΔΔCt^ relative quantitation method (Livak and Schmittgen, 2001). Acetate concentrations were also calculated as fold-change relative to control group (experimental or control value/mean control value) so that plasma and brain acetate changes could be compared to brain ACC levels. All western blot OD ratios, and QPCR and acetate fold changes were analysed using one way ANOVA and Tukey *post-hoc* tests.

## 3 Results

### 3.1 Effect of B-GOS^®^ intake on cortical NMDA responses

**Fig. 1:**
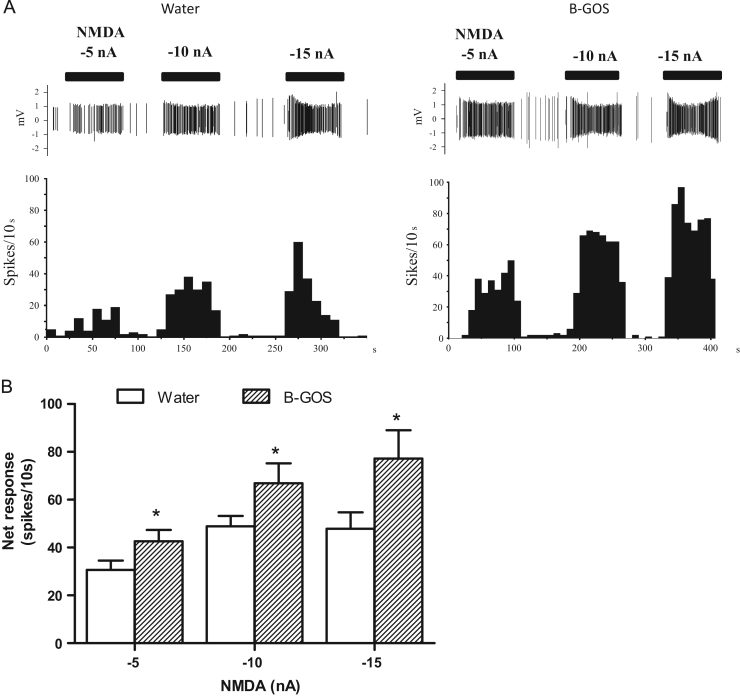
The effects of B-GOS supplementation on NMDA iontophoresis in the prefrontal cortex. (A) Burst activities (top panel) and firing rate histograms showing the individual responses to the iontophoretic application of NMDA, in two representative cortical neurons from the water and the B-GOS group. Each top horizontal bar represents the pulsed current applied onto the neuron that induced transient neuronal activation. (B) Cortical mean neuronal responses to iontophoretically applied NMDA at −5, −10 and −15 nA, in B-GOS-fed and control animals. There were no group x current interactions, but a significant effect of group where mean neuronal responses were greater in B-GOS rats compared to controls at all currents (nA). *p<0.05 compared to controls. *n*=36 (controls); *n*=44 (B-GOS).

In the first iontophoresis experiment, a total of 36 and 44 neurons in the control and B-GOS^®^ groups, respectively, were included (6–10 neurons per rat tested). All neurons tested exhibited the electrophysiological characteristics of pyramidal neurons according to pre-determined criteria (see above). Neurons from both B-GOS^®^ and water groups had similar low basal firing activity (12.6±2.6 and 7.7±1.2 Spikes/10 s for B-GOS^®^ and water groups, respectively, *p*>0.05), and more than 60% of the tested neurons from both groups had virtually no basal firing activity (firing rate <4 spikes/10 s). Iontophoretic application of NMDA, applied at currents between −5 and 10 nA, (Fig. 1A) produced a current dependent increase in firing activity, from baseline levels by 10–30 additional spikes/10 s per 5 nA of NMDA applied. However, the response plateaued at the highest current tested (−15 nA), being only slightly higher than the response obtained at −10 nA. This effect is probably due to saturation of NMDA receptors and/or partial depolarisation-inactivation, as some neurons tend to depolarise when currents higher than −10 nA were applied. Only neurons which produced consistent neuronal activations at a given current over time were selected. At all currents tested, the mean neuronal responses to iontophoretic NMDA in the PFC were greater in B-GOS^®^ -fed rats relative to controls (*F*_1,78_=7.01, *p*=0.011) (Fig. 1B). There was no diet x current (NMDA) interaction (*F*_2, 156_ = 0.99, *p*>0.05).

**Fig. 2:**
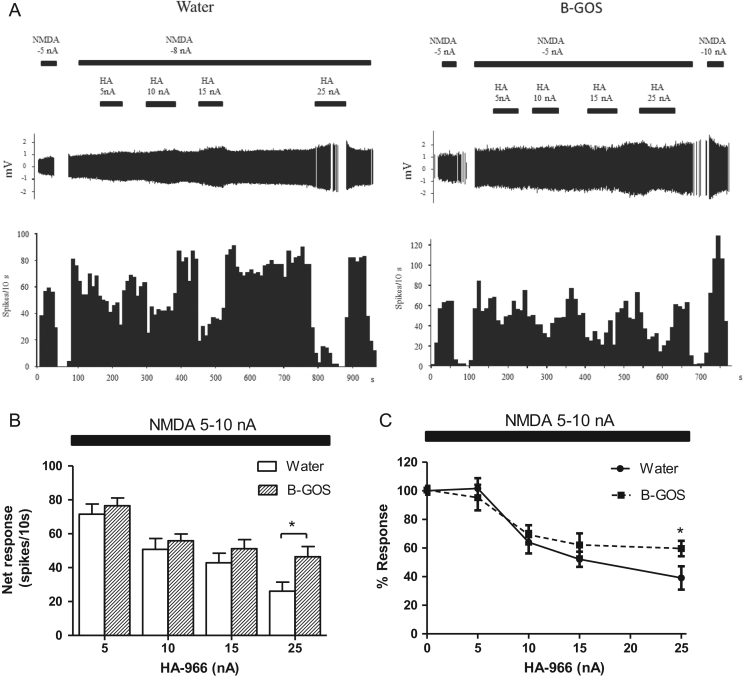
Cortical NMDA responses to HA-966 in B-GOS fed rats and controls. (A) Individual activity traces (top panel) and firing rate histograms in response to iontophoretic applications of NMDA and HA-966, of two representative neurons from each group. The figure shows that HA-966 more potently reduced the firing activation elicited by NMDA in the neuron from the control group compared to the neuron from the B-GOS fed rats. The NMDA pulses applied in the absence of HA-966 at the end of the experiment, demonstrated that neuronal responses were not influenced by preceding antagonist applications. (B) Burst activity traces (top panel) and mean responses to iontophoretically applied HA-966, at a fixed current of NMDA (typically between 5–10 nA, enough to produce a stable 4–5 HZ neuronal activation), decreased in both B-GOS and control rats. At −25 nA of HA-966, the NMDA response was less efficiently reduced in B-GOS rats compared to controls. (**C**) Inhibitory dose response curves showing the effect of HA-966 on NMDA cortical responses in water and B-GOS rats. *p<0.05, univariate analysis of group effect in 2-Way ANOVA (repeated measures); *n*=16/group.

In another experiment, the ability of the NMDAR glycine site antagonist, HA-966, to reduce the response of neurons to NMDA was tested. A long lasting application of NMDA (>10 min) at low currents (−5 to 10 nA), was sufficient to produce a stable level of mild activation (40–75 spikes/10 s) during which HA-966 was applied at various currents at 30–50 s intervals (Fig. 2). The iontophoretic application of HA-966 to cortical neurons inhibited NMDA-mediated responses at all currents tested (−5 to 25 nA), in a current-dependent manner in both B-GOS^®^ and control animals (Fig. 2A). A diet x current (HA-966) interaction was not observed (*F*_3,90_=1.15, *p*>0.05), although there was an effect of group (*F*_1,30_=4.91, *p*=0.037). This was driven by the greater magnitude of the NMDA-mediated response in B-GOS^®^ rats at −25 nA HA-966 compared to controls (*p*=0.013) (Fig. 2B). These data when plotted as % Response, (net response with HA-966/net response no HA-966 *100), confirmed that B-GOS^®^ reduced responses at −25 nA without shifting the inhibitory dose response curve relative to the water group (Fig. 2C).

**Fig. 3:**
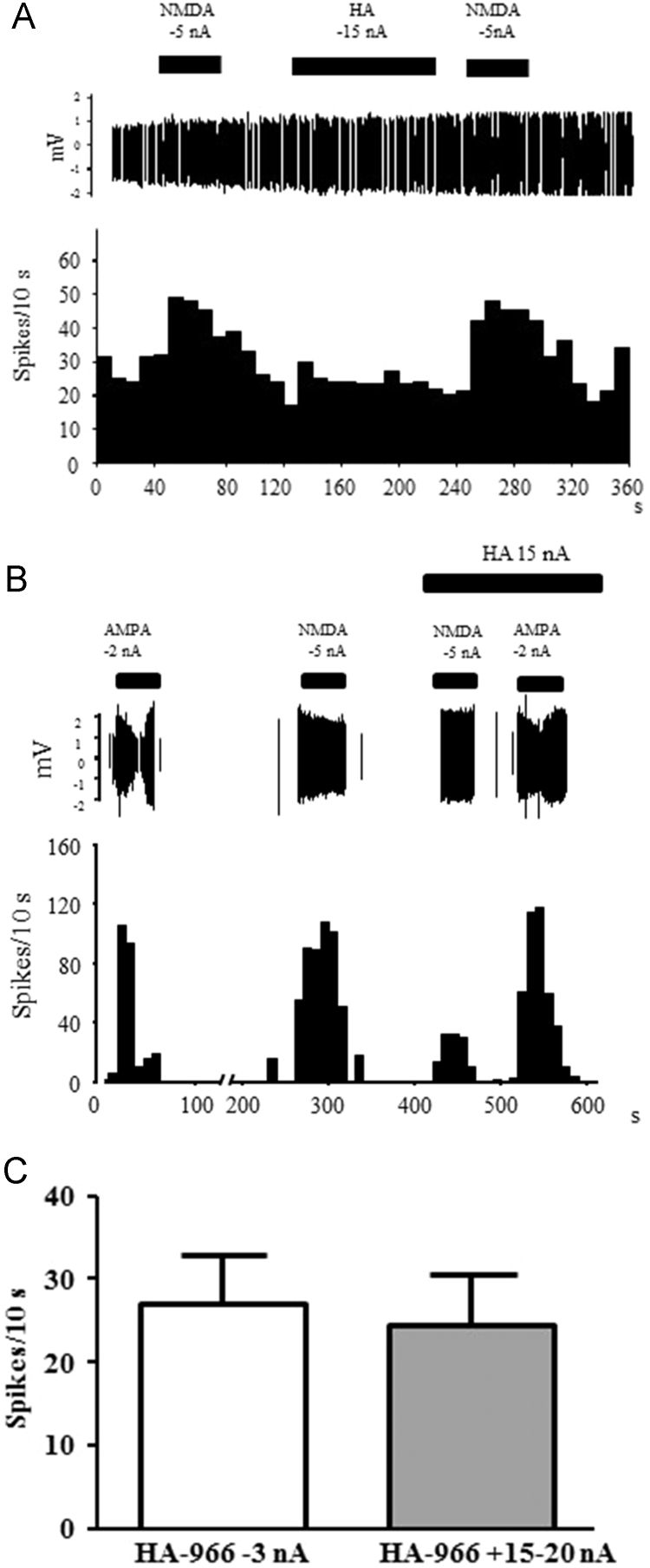
Neuronal responses to HA-966 alone and with iontophoretic application of NMDA and AMPA. (A) Representative traces (top panel) and neuronal response profile showing that application of HA-966 does not affect basal activity or subsequent NMDA responses after terminating its application. The latter confirms that HA-966 removal is rapid when its application is stopped. (B) Representative electrophysiological profile showing that HA-966 iontophoresis inhibits NMDA, but not AMPA, neuronal responses at 15 nA. (C). High concentrations of HA-966 applied to neurons (at similar current intensities used for inhibition of NMDA responses) does not affect neuronal activity in the cortex compared to controls (−3 nA, *n*=17).

To test whether HA-966 interacted with sites other than the NMDAR glycine binding site, neurons were exposed to the inhibitor in the absence of NMDA stimulation (Fig. 3). Histograms in Fig. 3A show that HA-966 applied at −15 nA did not affect baseline responses. The uninterrupted administration of NMDA before and after HA-966 application, revealed full recovery of the NMDA response after the cessation of antagonist application, and so confirmed that the selected neurons were fully responsive to NMDAR stimulation and that this response was sensitive to HA-966. Although neurons were also responsive to AMPA, AMPA-dependent activity was not blocked by HA-966 (Fig. 3B). This lack of effect of HA-966 toward AMPA-induced activity verified the specificity of the effect of HA-966 for NMDAR.

### 3.2 Effect of B-GOS on ASST performance, body weights, fluid intake and faecal *Bifidobacterium Spp.*

**Fig. 4:**
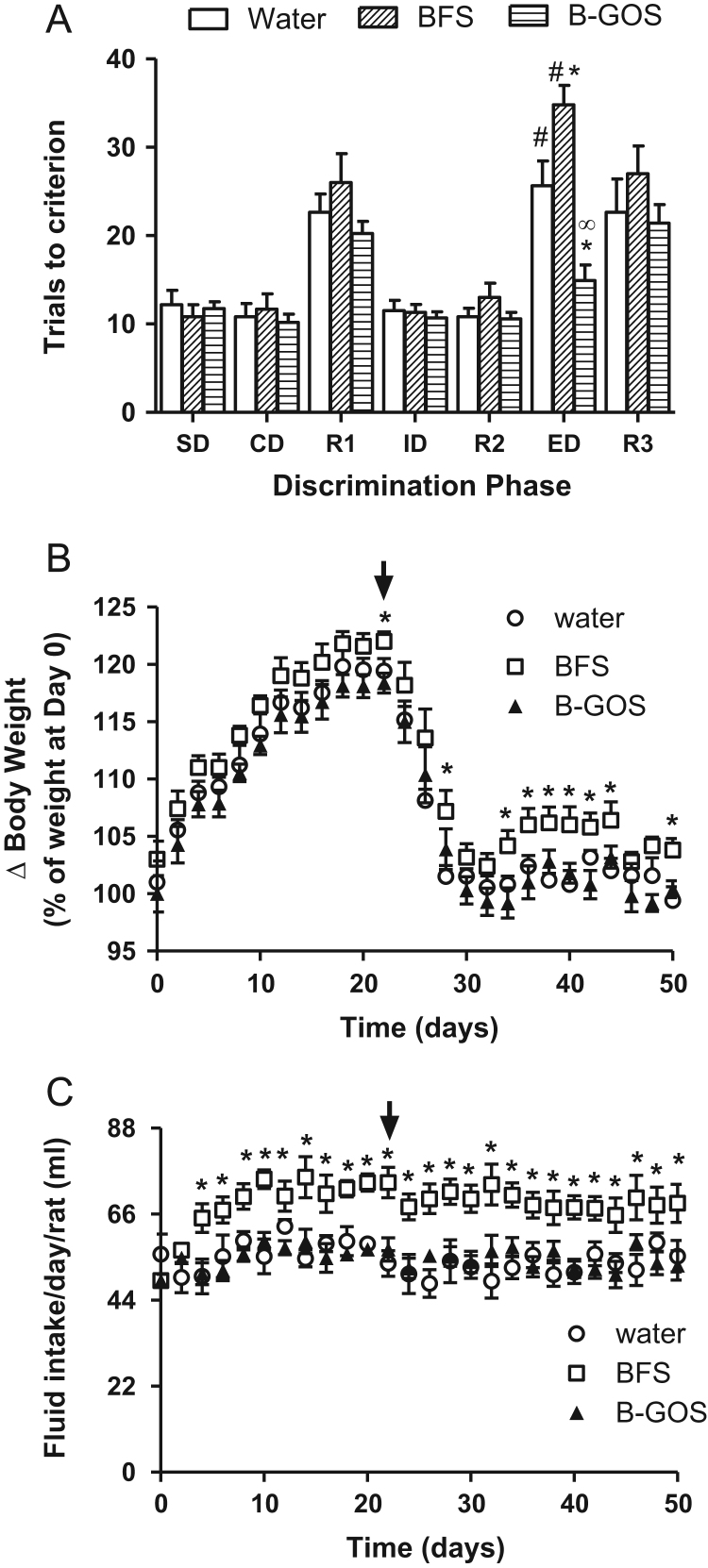
Attentional set-shifting performance, body weight and fluid intake in water, BFS and B-GOS rats. (A) The intake of B-GOS reduced the number of trials required to complete the ED phase to criterion, compared to controls. The significant difference between the number of trials in ID and ED seen with control rats, was not observed for B-GOS fed animals. Rats supplemented with BFS required more trials to criterion for ED compared to animals that had received standard drinking water or B-GOS. (B) Body weight of BFS-fed rats were significantly greater than controls during attentional-set shifting tasks. Arrow indicates the start of food restriction to obtain 85% of body weight for task performance. (C) Fluid intake by BFS-fed rats was significantly greater than controls 4 days after the start of supplementation and throughout the study. All data were analysed with 2-Way ANOVA (repeated measures). *p<0.05 compared to controls; #*p*<0.001 compared to ID phase; ∞ *p*<0.001 compared to BFS; *n*=12/group.

The administration of B-GOS^®^ improved ID/ED set shifting in the ASST paradigm, whereas rats provided with BFS performed worse than rats provided with normal drinking water (Fig. 4A). A significant diet × discrimination interaction (*F*_12,162_=4.43, *p*=0.001) was observed which was attributable to the effect of both B-GOS^®^ and BFS on ED performance relative to controls (*F*_2,27_=24.78, *p*<0.001). *Post hoc* analysis with Bonferroni correction revealed that B-GOS^®^ -fed rats required fewer trials to reach criterion in the ED discrimination task compared to controls (*p*=0.003), whereas the BFS supplemented rats required significantly more trials to reach criteria in the ED phase compared to both control (*p*=0.048) and B-GOS^®^ (*p*<0.001) animals. A significant difference between ID and ED performance in control (*F*_1,24_=30.49, *p*<0.001) and BFS (*F*_1,24_=83.91, *p*<0.001) groups was not observed for B-GOS^®^ rats (*F*_1,24_=2.03, *p*>0.1), and indicated improved ID/ED set-shifting, thus cognitive flexibility, in these animals.

The analysis of average changes in body weight throughout the supplementations and duration of the behavioural experiments did not reveal a diet × weight × time interaction, but showed a significant effect of diet (F_50,702_=49.04, *p*<0.001). This was driven by the elevation of body weight in BFS rats relative to controls (*p*<0.01) primarily on days 34–50, following food deprivation for ASST experiments (Fig. 4B). No significant differences in body weight were observed between controls and B-GOS^®^ rats (*p*>0.05). The analysis of fluid intake also revealed a significant effect of group (F_50,702_=173.98, *p*<0.001), which was attributed to the BFS rats drinking significantly more than controls (*p*<0.001) from days 4–50 (Fig. 4C). The intake of B-GOS^®^ supplemented water was not different to the consumption of water alone (*p*>0.05).

The QPCR analysis of *Bifidobacterium Spp.* in faecal DNA from rats subjected to ASST revealed a significant difference between groups (F_2,21_=5.043, *p*=0.021). The fold-change in bacteria in the B-GOS fed animals was approximately two-fold greater than the other groups (Means±SEM of Water=0.99±0.36; BFS=0.90±0.30; B-GOS^®^=2.45±0.47; *p*<0.05, Water vs B-GOS^®^ and BFS vs B-GOS^®^ groups).

### 3.3 Effects of B-GOS^®^ or GTA supplementation on GluN subunits, plasma and cortical acetate, and cortical ACC, HDAC1 and HDAC2 mRNAs

**Fig. 5:**
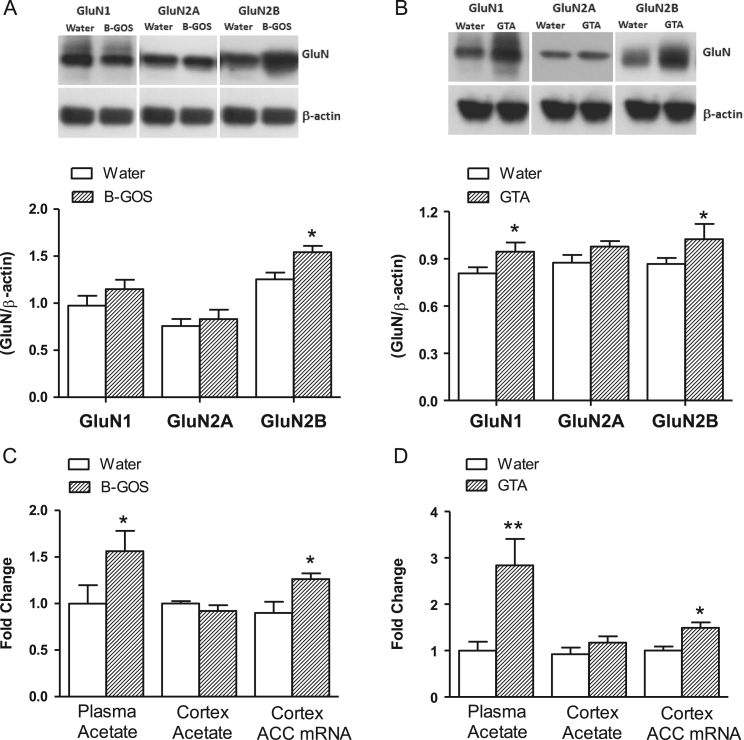
Effect of B-GOS or glyceryl triacetate (GTA) supplementation on rat cortex GluN subunits, acetate concentrations and acetyl Co-A carboxylase (ACC) gene expression. (A) Western blots demonstrated increased levels of cortical GluN2B subunits in B-GOS supplemented rats compared to controls. (B) A daily, 3 week administration of GTA (3 g/kg) increased cortical GluN1 and GluN2B levels. (C) In B-GOS fed rats, plasma acetate levels and cortical ACC gene expression were increased relative to controls, whereas cortical acetate levels remained unaltered. (D) GTA supplementation elevated plasma, but not brain, acetate concentrations and ACC mRNA. *p<0.05 compared to controls; *n*=8/group.

Western blot analysis of cortical tissue from rats (Fig. 5A) demonstrated a significant elevation of GluN2B in the B-GOS^®^ group compared to controls (*F*_1,20_=6.13, *p*=0.036). To explore the possibility that increased circulating acetate could influence NMDAR subunit expression, GluN subunits were measured in rats gavaged daily with GTA for 3 weeks. A significant increase of cortical GluN1 (*F*_1,20_=8.32, *p*=0.018), and GluN2B (*F*_1,20_=6.32, *p*=0.033) subunits were observed in GTA fed rats compared to controls (Fig. 5B).

Plasma acetate concentrations (*F*_1,20_=6.98, *p*=0.019) and the abundance of cortical ACC mRNA (*F*_1,20_=6.7, *p*=0.021), were also elevated by B-GOS^®^ supplementation, (Fig. 5C). The levels of cortical acetate did not alter with B-GOS^®^ feeding relative to controls. Similarly, cortical ACC mRNA abundance increased (*F*_1,20_=8.83, *p*=0.01), following GTA supplementation, and this was paralleled by an approximately 3-fold elevation of plasma acetate levels (*F*_1,20_=13.24, *p*=0.006), (Fig. 5D). No changes in cortical acetate levels were observed in these rats.

**Fig. 6:**
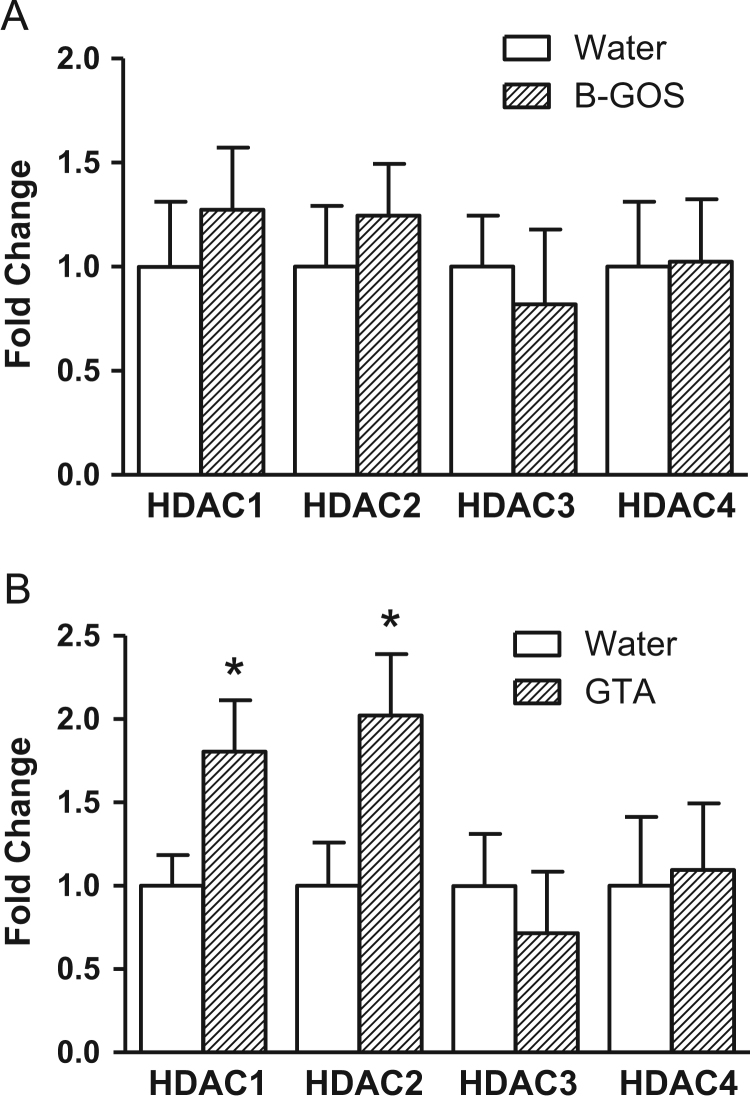
Effect of B-GOS or glyceryl triacetate (GTA) supplementation on rat cortex HDAC1-4 mRNAs. (A) B-GOS supplementation did not affect the expression of HDAC genes. (B) A daily, 3 week administration of GTA (3 g/kg) increased cortical HDAC1 and HDAC2 mRNAs, but not HDAC3 and HDAC4. *p<0.05 compared to respective controls; *n*=8/group.

The intake of B-GOS^®^ for 3 weeks did not affect HDAC (1–4) gene expression in the frontal cortex (Fig. 6A). However, GTA administration elevated both HDAC1 (*F*_1,20_=5.01, *p*=0.049) and HDAC2 (*F*_1,20_=5.09, *p*=0.048) mRNAs (Fig. 6B). Cortical HDAC3 and HDAC4 mRNA abundance remained unaltered following GTA supplementation.

## 4 Discussion

Maintaining healthy cognitive function has become one of the major challenges, both for neuropsychiatric disorders and healthy ageing in which optimal activity of the NMDAR is crucial. We have previously demonstrated that B-GOS^®^ intake by adult rats elevated cortical GluN1 subunits and its co-agonist, D-serine (Savignac et al., 2013). The current study investigated the functional correlates of these alterations by measuring cortical NMDAR responses and cognitive flexibility in B-GOS^®^ fed rats, and found increased neuronal responses to iontophoretically applied NMDA, and improved ID/ED set shifting in B-GOS^®^ supplemented animals compared to controls. Additional experiments suggest that acetate, a metabolite of B-GOS^®^ fermentation, might be involved in the elevation of NMDAR subunits following prebiotic intake. Overall, these data confirm that B-GOS^®^ influences NMDAR function, though the interpretation some specific findings require careful consideration.

The increased magnitude of NMDA-mediated responses following B-GOS^®^ intake compared to controls in the PFC, suggests elevated cortical NMDAR function. Our current observations, therefore, are in-keeping with our previous finding of increased GluN1 subunits in the cortex of B-GOS^®^-fed rats (Savignac et al., 2013). Since in that study, B-GOS^®^ feeding also elevated cortical D-serine, and in other work increased synaptic concentrations of NMDAR co-agonists hindered the dampening effects of HA-966 on NMDAR activity in rats (Chen et al., 2003), we tested if B-GOS supplementation attenuated HA-966 blockade. The lack of a B-GOS^®^ x HA-966 interaction, indicated that the antagonist reduced the NMDA-evoked responses in both controls and B-GOS^®^-fed rats, and suggests that prebiotic supplementation did not hinder HA-966 binding to the NMDAR. However, B-GOS^®^ did influence the magnitude of the NMDA response at the highest concentration of HA-966 (25 nA, Fig. 2B), relative to controls. Plotting these data as an inhibition dose response curve (Fig. 2C) not only confirmed the significant B-GOS^®^ effect at the highest concentration of inhibitor, but also revealed that both the prebiotic and control inhibition curves overlapped at the lower HA-966 concentrations. This argues against additional populations of GluN1 subunits or higher D-serine levels following B-GOS^®^ intake in the present investigation.

Increased levels of GluN1 subunits and/or D-serine relative to controls would be expected to cause a right-ward shift in the HA-966 inhibitory curve because, higher concentrations of the antagonists would be required to occupy the additional subunits and/or compete with higher synaptic concentrations of D-serine, and since this was absent after B-GOS^®^ intake the elevation of cortical GluN1 subunits (or D-serine) in this study can be ruled out. Indeed, western blots of rat cortical tissues revealed that the B-GOS^®^ administration increased GluN2B subunits compared to controls (Fig. 5A). This suggests elevated NMDAR function via increased GluN2B-containing populations of receptors and may explain a group difference at the highest HA-966 concentration. That is, lower concentrations of HA-966 may have been blocking a majority of NMDARs comprising of GluN2A subunits which are also abundant in the cortex, but when these became saturated the observed NMDA responses arose from the GluN2B containing receptors, which have different (slower) functional kinetics to the GluN2A moieties (Monaco et al., 2015). Of course, the differential responses between B-GOS^®^ and control groups may also suggest that the high antagonist concentrations might be binding to extraneous non-NMDAR sites. However, the demonstration that neither HA-966 alone nor in the presence of other excitatory stimuli (AMPA, Fig. 3) influence neuronal responses, confirms that the inhibitor was only targeting NMDARs. Future work into the mechanisms of B-GOS^®^ central actions might explore the involvement of other neurotransmitter systems such as GABA, which have been implicated in both the mediation of microbiome-gut-brain communication (Sarkar et al., 2016) and the modulation of cortical neural activity (Puig et al., 2005).

It is clear from the current electrophysiological experiments that B-GOS^®^ ingestion elevates cortical NMDAR function in rats, and from our previous studies, this does not seem to be mediated by changes in the concentration of peripheral or central glutamate itself (Savignac et al., 2013). However, the initial changes on which our present hypothesis was based ie elevated cortical GluN1, was not observed. One possible explanation is the method of prebiotic administration where in our original experiments rats were gavaged, but in the present study B-GOS^®^ was provided via their drinking water. The gavage technique is known to be stressful (Gonzales et al., 2014), and although control animals received the same manipulation in our earlier investigation, the procedure may have affected pre-existing levels of NMDAR subunits. The expression of total GluN1 is significantly reduced by stress (Yuen et al., 2012) and so if this occurred in our first study (Savignac et al., 2013) and B-GOS^®^ attenuated this effect, it would have been observed as an increase in these subunits relative to controls. This of course is speculative and the possibility that specific prebiotic feeding regimens lead to differential neurobiological outcomes would require further investigation.

Our data provide further evidence for the cognitive-enhancing effects of a non-pharmacological intervention that is known to proliferate beneficial gut bacteria (see also Burokas et al., 2017). The improvement in attentional set-shifting is consistent with our demonstration of improved attentional vigilance in healthy volunteers supplemented with B-GOS^®^ (Schmidt et al., 2015), and suggests that the prebiotic has a significant influence on cortical-mediated processes. Other evidence for the influence of beneficial gut bacteria on cortical function and cognitive processes has also been provided by some probiotic studies (Steenbergen et al., 2015, Tillisch et al., 2013, Carlson et al., 2017). We have also considered how the sugar content of B-GOS^®^ may have influenced behaviour. Studies subjecting rodents to tests of hippocampal function have reported poor spatial memory following a daily, 4 week administration of a 10% sucrose solution which also increased fluid intake (Kendig et al., 2013). Since the B-GOS^®^ solution provided to the rats contained only 0.42% glucose (or 1.53% of total sugars, see Experimental procedures), it seems unlikely that the intake of the sugars in the B-GOS^®^ formulation would influence behaviour at all. However, it is clear from our behavioural experiments that B-GOS^®^ sugars (BFS) impaired performance on the attentional set-shifting task (Fig. 4A). In this instance, the increased fluid intake with BFS, probably due to increased palatability of the solution in the absence of oligosaccharides, may have induced satiety and impaired the drive to find the food reward. Whatever the reason, it would appear that BFS is not an appropriate control for B-GOS^®^ when provided in the drinking water.

The B-GOS^®^ induced augmentation of cortical NMDAR electrophysiological responses, and improved set-shifting performance, provides further compelling evidence for the influence of supplements that proliferate beneficial gut bacteria, on central glutamate neurotransmission and their pro-cognitive effects. A deficit in attentional set-shifting abilities (behavioural responses to changes in environmental cues), is observed in schizophrenia (Pantelis et al., 2009), a disorder that has been associated with NMDAR hypofunction. In rats, the central NMDAR has been shown to be crucial for normal ASST performance, since its pharmacological blockade (Neill et al., 2010) or age-related reduction in the brain (Nicolle and Baxter 2003), hinders an animal's capacity to readily shift between two dimensions in the discrimination task. Improved ID/ED set-shifting in experimental models of NMDAR-impairment, has been achieved with nicotinic acetylcholine receptor agonists (Wood et al., 2016), or an opioid antagonist (Wallace et al., 2014), but the direct influence of increased NMDAR function on ASST performance in healthy rats has not been reported.

The next part of the study was to test the theory that the SCFA, acetate, a major microbial metabolite of B-GOS^®^ (Grimaldi et al., 2016), is involved in the modulation of cortical NMDAR levels. This was based on a recent report showing that acetate supplementation attenuated the molecular effects of NMDAR blockade by MK801 (Singh et al., 2016). We have demonstrated for the first time that B-GOS^®^ supplementation elevated circulating acetate concentrations and the cortical expression of ACC mRNA, which parallels the observed changes in these parameters following GTA administration. Importantly, since both interventions also elevated cortical GluN2B levels it is very plausible that acetate is a mediator of the psychotropic actions of B-GOS^®^. The additional elevation of GluN1 after GTA feeding, albeit also observed after B-GOS^®^ in an earlier study (Savignac et al., 2013), may either be related to the method of administration as discussed above, and/or the relatively greater levels of acetate arising from its direct administration. The latter possibility may also explain the differential effects of B-GOS^®^ and GTA on the expression of brain HDACs, but the key implication of these data is that epigenetic changes are not essential for B-GOS^®^-mediated central effects.

Acetate supplementation has been shown to elevate brain levels of Acetyl-CoA (Bhatt et al., 2013) and may have been the cause of elevated ACC gene expression following B-GOS^®^ and GTA intake ie increased ACC is a homeostatic response to normalize levels of its substrate generated from free acetate. However, the concentration of brain acetate is also modulated by its uptake, which can be hindered by NMDARs (Hirose et al., 2009). Therefore, our observed elevation of cortical GluN2B levels and NMDAR function after B-GOS^®^ feeding could also be interpreted as a homeostatic response to elevated circulating acetate. Thus, the reduced uptake of acetate through elevated NMDAR activity together with increased levels of ACC might explain the unaltered levels of brain acetate after B-GOS^®^ (Fig. 5B) or GTA (Fig. 5D).

Indirect evidence for increased central metabolism underlying the actions of B-GOS^®^ on the brain was the finding that HDAC gene expression remained unaltered after prebiotic feeding, which excluded the involvement of acetate-mediated epigenetic effects. We have confirmed the elevation of HDAC1 and HDAC2 following GTA (Soliman et al., 2012, and see Fig. 6) at the level of encoding mRNA which supports data showing that direct acetate supplementation does influence central epigenetic pathways. It is noteworthy that GTA-mediated attenuation of MK801 effects, involved increased brain histone acetylation and activation of NMDAR-independent signalling. Our current finding may indicate, therefore, that circulating acetate levels following B-GOS^®^ ingestion activate NMDAR mediated processes whereas the higher concentrations that arise from GTA supplementation, trigger additional epigenetic processes. This interpretation of our findings would require testing by performing GTA dose response experiments and measuring NMDAR function and brain histone acetylation. Furthermore, changes in HDAC activity would also be important to assess since this may alter independently of encoding mRNA eg via post-translational phosphorylation (Singh et al., 2016).

It should be emphasized that this study only focussed on the involvement of acetate because it is a major metabolite of B-GOS^®^ and has been linked to NMDARs (Hirose et al., 2009, Singh et al., 2016). Moreover, the detection of circulating acetate is easily achieved using rapid, commercially available kits that can be used for subsequent human trials. Of course, acetate is not the only SCFA that results from microbial fermentation of B-GOS^®^. Increased enteric butyric acid following B-GOS^®^ intake (Grimaldi et al., 2016, Grimaldi et al., 2017) has significant effects on the gut epithelium and may influence central functions via the established routes (eg immunity) of the gut-brain axis (Stilling et al., 2016). Moreover, butyrate directly affects central gene expression via epigenetic mechanisms (Han et al., 2014, Stilling et al., 2016). Thus, the effect of this SCFA on brain HDAC activity/protein phosphorylation status following the administration of butyrate would have to be analysed to completely rule out the role of this metabolite in the central actions of B-GOS^®^.

Finally, it is important to note that a recent study using another formulation of GOS and in combination with a fructo-oligosaccharide (FOS), demonstrated psychotropic effects but did not reveal an association between acetate levels and NMDAR subunits in the hippocampus (Burokas et al., 2017). In the light of our present data, this may suggest that acetate may have region specific effects. However, the study also demonstrated a reduction of *Bifidibacteria* and *Lactobacilli* with the FOS and GOS formulations whereas we have reported the increase in the former with B-GOS^®^ (Savignac et al., 2013, Savignac et al., 2016); this indicates that even the primary actions of these prebiotics are different. Of course, it would be naïve to conclude that the modulation of brain function solely depends on changes in the levels of just two genera of bacteria given the vastness of the gut microbiome, but the discrepancies do emphasise that our findings with B-GOS^®^ are only applicable to this specific prebiotic mixture, and cannot be extended to all formulations of GOS. The current study used B-GOS^®^ because it is readily available to the consumer (Bimuno^™^, for composition see Vulevic et al., 2008), and its bifidogenic actions in animals and humans have been well documented (see above). Future mechanistic and functional studies with other prebiotics therefore, will need to consider not only animal species and appropriateness of tests for different brain regions, but also the formulation and principal effect of the compound under investigation.

In conclusion, the current study provides an original demonstration of elevated cortical NMDAR responses and improved cognitive flexibility following B-GOS^®^ intake. A potential mechanism has also been proposed where increased circulating acetate that results from B-GOS^®^ metabolism may affect NMDAR neurobiology via metabolic pathways rather than through direct influences on epigenetic mechanisms that modulate gene expression. Supplementing rats directly with acetate provided this initial proof-of-concept, but additional experiments are required to confirm whether changes in NMDAR subunit levels after GTA have similar functional actions as B-GOS^®^ feeding. One important implication of our findings is that the ingestion of B-GOS^®^ in adulthood may offer ‘neuroprotection’ and limit age-related deterioration of NMDAR and cognitive function. Another possibility is that B-GOS^®^ might be used as an adjunctive therapy in neuropsychiatric disorders such as schizophrenia, depression, or dementia, where cognitive deficits are a prominent feature of these illnesses, and do not respond to current treatments. Human trials are therefore required to validate these possibilities, and further explorations into the molecular mechanisms underlying the central effects of B-GOS^®^, may reveal additional pathways through which gut bacteria influence brain function synergistically with the metabolites of this prebiotic.

## Role of funding source

This work was funded by a Biotechnology, Biological Sciences Council (BBSRC) Industrial Partnership Award (Principal Investigator, Dr Burnet; Grant Code: BB/I006311/1). Clasado Biosciences Ltd., provided a 10% financial contribution towards the study (Grant Code: HQRTZB1), as part of the BBSRC scheme. Neither the BBSRC nor Clasado Biosciences Ltd., had any further role in the study design, the collection, analysis and interpretation of data, the writing of the report, and in the decision to submit the paper for publication.

## Contributors

Drs Gronier, Savignac and Burnet made a substantial contribution to the conception and design of the study, analysis and interpretation of the data together with Dr Di Miceli, Mr Idriss and Prof Anthony. Drs Burnet, Gronier, Savignac and Prof Anthony drafted the article, which was reviewed for intellectual content by Dr Tzortzis.

## Conflict of interest

Dr Tzortzis is an employee of Clasado Biosciences Ltd. Dr Savignac is an employee of 4D Pharma.
